# Neutrophils Are Dysregulated in Patients with Hereditary Angioedema Types I and II in a Symptom-Free Period

**DOI:** 10.1155/2019/9515628

**Published:** 2019-05-19

**Authors:** Tereza Grymova, Marcela Vlkova, Premysl Soucek, Roman Hakl, Jana Nechvatalova, Peter Slanina, Julie Stichova, Jiri Litzman, Tomas Freiberger

**Affiliations:** ^1^Centre for Cardiovascular Surgery and Transplantation, Brno, Czech Republic; ^2^Department of Clinical Immunology and Allergology, Faculty of Medicine, Masaryk University, Brno, Czech Republic; ^3^St. Anne's University Hospital, Brno, Czech Republic; ^4^Central European Institute of Technology (CEITEC), Masaryk University, Brno, Czech Republic

## Abstract

Neutrophils impact on processes preceding the formation of bradykinin, a major swelling mediator in hereditary angioedema (HAE), yet their potential role in HAE pathogenesis has not been sufficiently studied. We assessed the relative mRNA expression of 10 genes related to neutrophil activation using RNA extracted from the peripheral blood neutrophils of 23 HAE patients in a symptom-free period and 39 healthy donors. Increased relative mRNA expression levels of *CD274*, *IL1B*, *IL1RN*, *IL8*, *MMP9*, and *TLR4*, together with a lack in their mutual correlations detected in HAE patients compared to healthy controls, suggested a preactivated state and dysregulation of patients' neutrophils. Patients' neutrophil-alerted state was further supported by increased CD11b, decreased CD16 plasma membrane deposition, and increased relative CD274^+^ and CD87^+^ neutrophil counts, but not by increased neutrophil elastase or myeloperoxidase plasma levels. As CD274 mediates inhibitory signals to different immune cells, neutrophils were cocultured with T-cells/PBMC. The decrease in CD25^+^ and IFN-*γ* ^+^ T-cell/PBMC ratio in patients indicated the patients' neutrophil suppressive functions. In summary, the results showed neutrophils' alerted state and dysregulation at the transcript level in patients with HAE types I and II even in a symptom-free period, which might make them more susceptible to edema formation. Neutrophils' T-cell suppressive capacity in HAE patients needs to be further investigated.

## 1. Introduction

Hereditary angioedema (HAE) is a rare autosomal dominant disorder manifesting recurrent submucosal and subcutaneous edema episodes. HAE can be caused by mutations in *SERPING1*, resulting in decreased C1 inhibitor level (type I) and/or impaired C1 inhibitor function (type I, type II), or by mutations in *FXII*, causing factor XII hyperactivity [1–5]. Recently, a mutation in *PLG* (encoding plasminogen) was reported to be associated with HAE [6]. These types of genetic alternations lead to uncontrolled kallikrein-kinin system activation and thus to excessive bradykinin generation, considering the major swelling mediator in HAE [7, 8]. Another newly described type of HAE is associated with a mutation in *ANGPT1* (encoding angiopoietin-1). ANGPT1 was shown to decrease plasma leakage induced by bradykinin [9].

Neutrophils are the most abundant leukocytes (50-70% of all circulating leukocytes). They are the first cells that accumulate at inflammatory sites. Their recruitment from the blood into the tissue, where they are fully activated, is orchestrated by various families of adhesion molecules, chemokines, and cytokines, i.e., Mac-1, LFA-1, ICAM-1, VCAM-1, MIP-2, interleukin- (IL-) 8, or C5a. Originally, neutrophils were considered to subserve the only, although pivotal, function that lies in microorganisms' phagocytosis. Nowadays, it is clear that the repertoire of their functions is much broader. Neutrophils are capable of eliminating microorganisms also in an extracellular space via neutrophil extracellular traps (NETs). Moreover, neutrophils are involved in initiating and modulating immune responses by releasing various cytokines and interactions with all major types of immune cells [10–12].

Several lines of evidence suggest that neutrophils exert multiple influences on the processes preceding bradykinin formation. Neutrophil elastase (NE), released from activated neutrophils, can cleave and inactivate the C1 inhibitor and thus allow kallikrein-kinin system activation [13]. Furthermore, neutrophil activation can lead to the formation of NETs that provide a negatively charged surface suitable for FXII autoactivation. On the surface of neutrophils, the kallikrein-kinin system can also be activated [14].

On the other hand, components of the kallikrein-kinin system modulate neutrophil functions. It was reported that the bradykinin B1 receptor regulates neutrophil migration *in vitro* [15]. Both the kallikrein and activated FXII can cause neutrophil degranulation [16].

Despite these findings, the potential role of neutrophils in HAE pathogenesis has not been studied adequately. Veszeli et al. observed increased plasma levels in selected proteins (NE, myeloperoxidase (MPO), pentraxin 3, and IL-8) potentially related to neutrophil activation in HAE patients during an attack compared to healthy donors, but not in HAE patients in a symptom-free period [17]. In the presented study, we investigated if neutrophils can be in an activated state even in a symptom-free HAE period. The aims of our study were as follows: (1) to assess relative mRNA expression of selected genes involved in neutrophil activation, degranulation, migration and neutrophil-mediated T-cell suppression; (2) to measure surface and plasma neutrophil activation marker levels; and (3) to evaluate the potential effect of neutrophils on PBMC/T-cells by determining ratios of CD25^+^ and IFN-*γ* ^+^ PBMC/T-cells after coculture with autologous neutrophils.

## 2. Materials and Methods

### 2.1. Patients and Healthy Donors

The study was approved by the Medical Ethics Committee of St. Anne's University Hospital (ethics approval number: 6G/2015, Brno). Informed consent was obtained from all the participants before being included in the study. The study conforms to the standards of the Declaration of Helsinki. A total of 23 HAE patients (20 type I and 3 type II; 10 females and 13 males) and 39 healthy donors (HD; 18 females and 21 males) were recruited. None of the patients displayed symptoms of HAE attack or infectious disease at the time of sampling nor suffered from chronic infectious complications. The median age of disease onset was 16 years (range: 11-30 years). The median period from first clinical symptoms to the diagnosis was 14 years (range: 1-33). One patient, but not one of the healthy donors, was a smoker. Nine patients were on a long-term prophylactic treatment: 5 using tranexamic acid and 4 using danazol. Complement levels, function, and treatment are listed in Supplementary table 1.

### 2.2. Cell Isolation

Peripheral Blood Mononuclear Cells (PBMCs) and neutrophils were isolated from the blood harvested in EDTA-containing vacutainers by density gradient centrifugation (400x g, 30 min) using Ficoll-Paque (Pharmacia) and washed twice with phosphate-buffered saline (PBS) supplemented with 5% bovine serum albumin (Sigma). Following centrifugation, the upper mononuclear cell layer was removed, and the lower granulocyte layer was collected and resuspended. For neutrophil isolation, techniques minimizing the stress associated with neutrophil enrichment, such as the use of isotonic red blood cell lysis buffer (NH_4_Cl erythrocyte lysis buffer: 170 mM NH_4_Cl, 10 mM KHCO_3_, 20 mM EDTA, pH 7.3; room temperature), were used [18]. The levels of neutrophil activation markers CD11b and PD-L1 after isolation were evaluated (Fig.  S2A). The cells were cultured in RPMI 1640 (Sigma) supplemented with 10% heat-inactivated fetal bovine serum (FBS) (HyClone), 100 U/mL penicillin, 100 mg/mL streptomycin, and 2 mM L-glutamine (HyClone) (complete RPMI medium). This procedure resulted in 95% neutrophil purity, as determined by CD15 marker expression.

T-cells were isolated from the peripheral blood using a RoboSep Human CD3 Positive Selection Whole Blood kit (STEMCELL Technologies). Separation was performed on a RoboSep TM-S instrument (STEMCELL Technologies) according to the manufacturer's protocol. The cell purity of the isolated T-cells was above 98%, as determined by flow cytometry.

### 2.3. RT-qPCR

The relative expression of 10 genes related to neutrophil activation (*CCL3*, *CD274*, *CXCL2, FASLG*, *IL1B*, *IL1RN*, *IL8*, *MMP9*, *TLR2*, and *TLR4*) was assessed using RT-qPCR. The total RNA was isolated using a *mir*Vana™ miRNA Isolation Kit (Roche) and reverse-transcribed using a Transcriptor First Strand cDNA Synthesis Kit (Roche). Real-time PCR was performed on a LightCycler® 480 Instrument II (Roche) using TaqMan® Gene Expression Assays (Hs01060665_g1, Hs00851655_g1, Hs00234142_m1, Hs01125301_m1, Hs00601975_m1, Hs00181225_m1, Hs00174097_m1, Hs00893626_m1, Hs00174103_m1, Hs00234579_m1, Hs01872448_s1, and Hs00152939_m1; Applied Biosystems). Obtained data were analyzed in the LightCycler® 480 SW 1.5.1 (Roche) using the Fit Points method and normalized to *ACTB* and *RPL32* housekeeping genes' expression.

### 2.4. Flow Cytometry of Whole Blood

To determine the neutrophil activation surface markers from the whole blood, mAbs were used in the following combinations: (1) CD15-FITC (clone HI98), CD80-PE (clone 2D10), CD14-PerCP-Cy5.5 (clone M5E2), CD274-APC (PD-L1) (clone 29E.2A3), and CD62L-BV421 (clone DREG-56) (BioLegend) and CD16-APC A750 (clone 3G8) and CD45-Krome Orange (KO) (clone J33) (Beckman Coulter); (2) CD15-FITC, CD87-PE (clone VIM5), CD14-PerCP–Cy5.5, CD11a-PE-Cy7 (clone HI111), HLA-DR- BV421 (clone L243), CD11b- BV 510 (clone ICRF44), (BioLegend), and CD45-APC-H700 (clone 2D1) (BD Biosciences). To determine Treg in lymphocyte populations, monoclonal antibodies (mAbs) were used in the following combinations: CD127-PE (clone R34.34), CD25 R Phycoerythrin-Cyanine 5.1 (clone B1.49.9), CD4-PE-Cy7 (clone SFCI12T4D11), CD45-Krome Orange (KO) (clone J33) (Beckman Coulter), and CD3-APC (clone UCHT1) (BioLegend).

First, the blood samples were incubated for 30 min at 4°C in the dark with mABs. Then, the erythrocytes were lysed by Multi-Q-Prep Lysing Workstation (Beckman Coulter). A Navios flow cytometer (10 colors, 3 lasers; Beckman Coulter) was used to analyze the samples. Whole blood neutrophils were gated as SSC^hi^CD45^+^CD15^+^ populations, and surface marker levels were evaluated (Fig.  S2B). Whole blood Treg cells were gated as CD45^+^CD3^+^CD4^+^CD25^+^CD127^low^ population. The obtained cytometry data (LMD files) were analyzed using Kaluza software (Beckman Coulter).

### 2.5. Neutrophil/T-Cell Suppression Assay

PBMC/T-cells were stimulated with 1 *μ*g/mL purified plate-bound anti-CD3 mAb (clone Hit-3a; BioLegend) and soluble 0.3 *μ*g/mL anti-CD28 mAb (clone CD28.2; eBioscence) for 18 hours in complete RPMI medium in 96 flat-bottom wells with a starting concentration of 2 × 10^5^ cells/well (1 × 10^6^/mL) of PBMCs or 1 × 10^5^ cells/well (1 × 10^6^/mL) of T-cells at 37° C, 5% CO_2_. For neutrophil/T-cell suppression assay, neutrophils were cocultured with CD3^+^ PBMC/T-cells at a 3 : 1 ratio at 37° C, 5% CO_2_.

CD25 surface expression was assessed using flow cytometry. The following mAbs were used for PBMC/T-cell staining: CD4-PE-Cy7 (clone SFCI12T4D11), CD8-APC A750 (clone B9.11) (Beckman Coulter), CD3-APC (clone SK7) (BD Biosciences), and CD25-PerCP-Cy5.5 (clone BC96) (BioLegend).

To detect IFN-*γ* by intracellular staining, brefeldin (10 *μ*g/mL) was added for the last 4 hours of incubation. The cells were washed, fixed, permeabilized using Intracellular Fixation and Permeabilization Buffer Set (eBioscience) according to the manufacturer´s protocol, and stained with IFN-*γ*-BV421 (clone 4S.B3) (BioLegend).

### 2.6. ELISA for Cytokine Detection

Both supernatants from cell cultures and plasma separated by centrifugation (1500x g, 4°C, 15 minutes) were cryopreserved at -80°C. IFN-*γ* in a supernatant and IL-8 in a plasma were measured by ELISA assay (BioLegend, San Diego, USA), according to the manufacturer's protocol.

### 2.7. Elastase and Myeloperoxidase Plasma Levels

NE and MPO plasma levels were determined by ELISA according to the manufacturer's protocol (Hycult Biotech, Plymouth Meeting, USA).

### 2.8. Statistics

If not mentioned otherwise, data in the patient/control groups are presented as the median. The Mann-Whitney *U* test was used to analyze the differences between the donor and patient group. The Wilcoxon signed rank test was used to assess neutrophil's effect on PBMC/T-cells. The correlations were examined using Spearman's rank correlation coefficient test. The Kolmogorov-Smirnov test was used to analyze the distribution of absolute neutrophil counts. *p* values < 0.05 were considered statistically significant. Data was analyzed using STATISTICA 12 and GraphPad Prism 5.

## 3. Results

### 3.1. mRNA Expression of Genes Related to Neutrophil Activation

**Figure 1:**
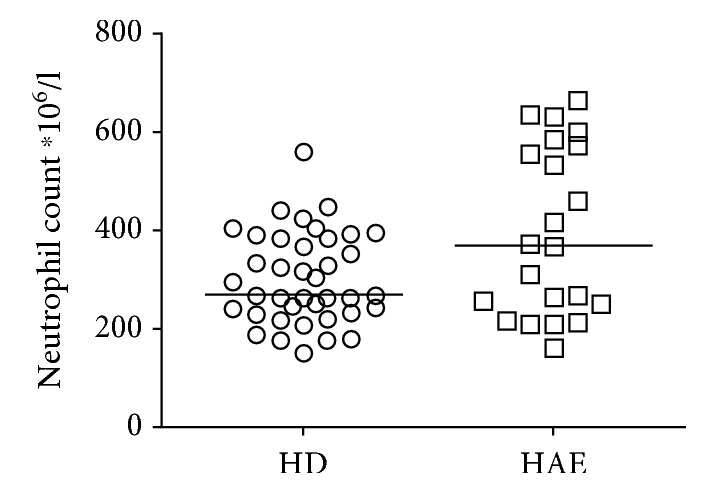
The absolute neutrophil count in healthy donors (HD) and HAE patients. Horizontal bars represent medians.

It was previously reported that the absolute number of neutrophils might increase during HAE attacks [19]. In one study, increased absolute neutrophil count was detected in HAE patients, even in a symptom-free period [17]. In our study, focused on HAE patients' neutrophils in a symptom-free period, no significant difference (Mann-Whitney *U* test) in the absolute neutrophil count was observed between the patients and healthy donors (HD) (Figure 1). Nevertheless, according to the Kolmogorov-Smirnov test, absolute neutrophil count distributions differed between HAE patients and HD (*p* = 0.042). Thus, it is evident that the absolute neutrophil count is increased in at least some of the patients.

**Figure 2:**
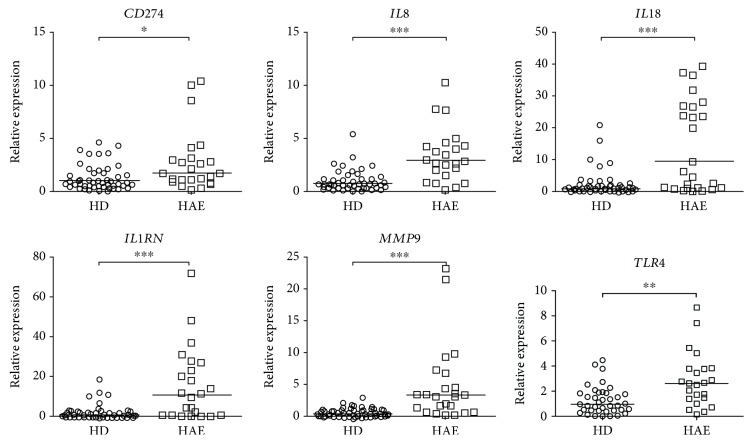
The relative mRNA expression of genes related to neutrophil activation in HAE patients and healthy donors (HD). Horizontal bars represent medians, and stars represent the statistical significance: ^∗^ *p* ≤ 0.05, ^∗∗^ *p* ≤ 0.01, and ^∗∗∗^ *p* ≤ 0.001.

While examining the genes involved in neutrophil activation, degranulation, migration, and neutrophil-mediated T-cell suppression, we found out that patients' neutrophils exerted an altered expression profile compared to the neutrophils obtained from HD. Relative *mRNA* expression analyses showed increased *CD274* (1.69 vs. 1.01, *p* = 0.038), *IL1B* (9.32 vs. 0.95, *p* = 0.0008), *IL1RN* (11.05 vs. 0.65, *p* = 0.0001), *IL8* (2.94 vs. 0.78, *p* < 0.0001), *MMP9* (3.33 vs. 0.45, *p* < 0.0001), and *TLR4* (2.62 vs. 0.96, *p* = 0.0012) levels in HAE patients than in HD (Figure 2). Relative *mRNA* expression levels of *CCL3*, *CXCL2*, *FASLG*, and *TLR2* did not differ in HAE patients compared to those in HD (Fig.  S1). These data suggest patients' neutrophils are in an alerted state at the transcript level in a symptom-free period.

### 3.2. Correlation between mRNA Expression Levels of Selected Genes

To address a potential relationship between genes with a relative mRNA expression level significantly differing between HAE patients and HD, correlation analyses were performed. In the group of HD, all genes except *IL1RN* positively correlated with each other. On the contrary, weaker or even no correlation was observed in most cases in the group of HAE patients compared to HD. Interestingly, positive correlation occurred between *IL8* and *IL1RN* in the patient group (Table 1).

### 3.3. Differences in the Expression Level of Neutrophil Surface Markers

**Figure 3:**
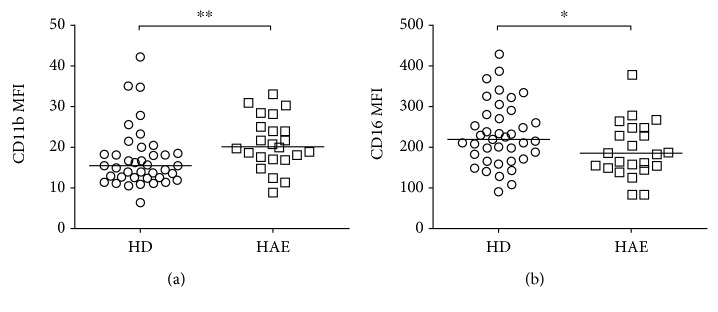
Expression levels of neutrophil surface markers in HAE patients and healthy donors (HD). Neutrophils were gated as CD45^+^CD15^+^CD16^+^ SSC^high^ cells. (a) Increased expression of CD11b and (b) decreased levels of CD16 on neutrophils in fresh blood of HAE patients. representative examples, and cumulative data. MFI: median fluorescence intensity. Horizontal bars represent medians, and stars represent the statistical significance: ^∗^ *p* ≤ 0.05 and ^∗∗^ *p* ≤ 0.01.

To further investigate neutrophil activation in HAE patients suggested by expression analyses, neutrophil surface markers were evaluated using flow cytometry comparing median fluorescence intensity (MFI). Whole blood neutrophils were gated as SSC^high^ CD45^+^CD15^+^ populations, and the surface levels of CD11a, CD11b, CD16, CD62L, CD80, CD87, PD-L1, and HLA-DR were examined. There was a significant increase in CD11b MFI (19.89 vs. 15.45, *p* = 0.0071) and a decrease in CD16 MFI (218.7 vs. 183; *p* = 0.0443) in HAE patients compared to HD (Figure 3, Fig.  S3), which supported neutrophil activation in HAE patients.

### 3.4. Differences in the Ratios of Neutrophils Expressing Specific Surface Markers of Activation

**Figure 4:**
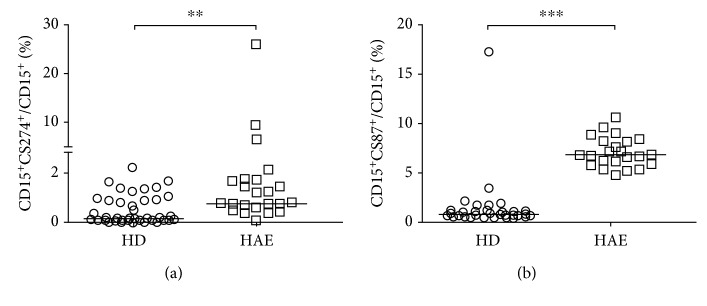
Ratios of neutrophils expressing surface activation markers. (a) Neutrophils expressing CD274. (b) Neutrophils expressing CD87. Horizontal bars represent medians, and stars represent the statistical significance: ^∗^ *p* ≤ 0.05, ^∗∗^ *p* ≤ 0.01, and ^∗∗∗^ *p* ≤ 0.001.

Specific neutrophil activation was examined by comparing the ratios of neutrophils expressing an appropriate marker. A significant increase in the relative CD274^+^ neutrophils count (0.79 % vs. 0.16 %, *p* = 0.0016) and CD87^+^ neutrophils (6.82 % vs. 0.91 %, *p* < 0.0001) in HAE patients compared to HD (Figure 4) further indicated neutrophil alertness in HAE patients. Moreover, the increased CD274^+^ neutrophil ratio suggested neutrophil-mediated T-cell suppression. On the other hand, no significant difference was observed in the CD80^+^ neutrophil ratio between HAE patients and HD (Fig.  S4).

### 3.5. Plasma Neutrophil Activation Markers

**Figure 5:**
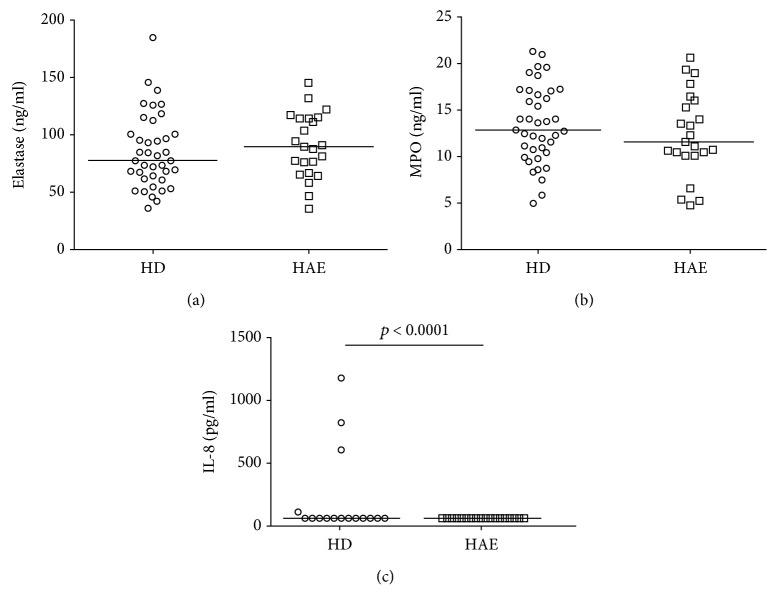
Myeloperoxidase (MPO) and elastase (NE) levels determined using ELISA were not significantly different between HAE patients (*n* = 20) and healthy donors (HD, *n* = 39), while interleukin- (IL-) 8 was significantly decreased in HAE patients (*n* = 23) compared to HD (*n* = 16). Horizontal bars represent medians. Data were analyzed using the Mann-Whitney *U* test.

As increased NE activity, MPO, and IL-8 release accompany neutrophil activation [20, 21], their plasma levels were assessed and reached 80.7 *μ*g/L of NE, 12.4 *μ*g/L of MPO, and 54 pg/mL of IL-8 in patients' plasma, which did not significantly differ from the healthy controls' levels in NE and MPO (NE: 75.4 *μ*g/L; MPO: 13.5 *μ*g/L; *p* > 0.05 in both cases), but was significantly decreased compared to the healthy controls' IL-8 levels in (58 pg/mL, *p* < 0.0001), using a Mann-Whitney test (Figure 5).

### 3.6. Tregs and Effect of Neutrophils on T-Cell Activation

Treg representation in lymphocyte populations was determined in 15 HAE patients and 23 controls, showing significantly increased Treg levels in patients compared to those in controls (3.9 % vs. 3.1 %, *p* < 0.02, using a Mann-Whitney test).

To functionally evaluate possible T-cell suppression mediated by CD274^+^ neutrophils, we investigated the neutrophils' effect on T-cell activation and cytokine production. Changes in surface CD25 expression and INF-*γ* production by CD3/CD28-stimulated T-cells after incubation with autologous neutrophils were assessed.

**Figure 6:**
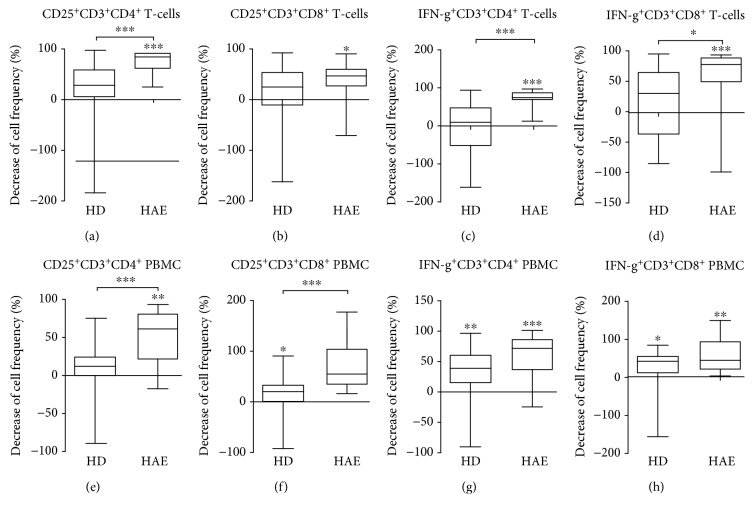
Decreases in CD25 expression/IFN-*γ* production on/in T-cell/PBMC of healthy donors (HD) and HAE patients after coculture with autologous neutrophils. (a, b) Coincubation of T-cells with autologous neutrophils in response to CD3/CD28 stimulation results in reduced percentages of CD25^+^ T-cells of total CD3^+^CD4^+^ or CD3^+^CD8^+^ T-cells in HAE patients. (c, d) Coincubation of T-cells with autologous neutrophils in response to the stimulation with CD3/CD28 results in reduced percentages of IFN-*γ* ^+^ T-cells of total CD3^+^CD4^+^ or CD3^+^CD8^+^ T-cells in HAE patients. (e, f) PBMC coincubation with autologous neutrophils in response to stimulating with CD3/CD28 results in reduced CD25^+^ T-cell percentages of total CD3^+^CD4^+^ cells in HAE patients and CD3^+^CD8^+^ T-cell in HD. (g, h) Coincubation of PBMC with autologous neutrophils in response to stimulation with CD3/CD28 results in reduced percentages of IFN-*γ* on CD3^+^CD4^+^ or CD3^+^CD8^+^ T-cells in HAE patients and HD too (horizontal bars represent medians; whiskers: 10-90 percentile; stars represent the statistical significance: ^∗^ *p* ≤ 0.05, ^∗∗^ *p* ≤ 0.01, and ^∗∗∗^ *p* ≤ 0.001.

Surface CD25 expression was considerably reduced on CD4^+^ T-cells after coculture with neutrophils in HAE patients (81.4 %, *p* < 0.0001), but not in HD (Figure 6(a), Fig.  S6A). In CD8^+^ T-cells, surface CD25 expression was also reduced after coculture with HD neutrophils in HAE patients (44.6 %, *p* = 0.0369) and not in HD, but the decreases between groups (HD vs. HAE) did not differ significantly (Figure 6(b), Fig.  S6B).

Similarly, neutrophils considerably reduced the ratio of IFN-*γ* producing CD4^+^ T-cells in HAE patients (74.6 %, *p* < 0.0001) and not in HD (Figure 6(c), Fig S6C). A similar effect was observed on IFN-*γ* production in CD8^+^ T-cells (73.82 % decrease in HAE patients, *p* < 0.0001) (Figure 6(d), Fig.  S6D).

To assess whether the neutrophil's suppressive effect can be negated by the monocytes' stimulating effect, the same experiment was also performed with PBMC.

Surface CD25 expression was considerably reduced on CD4^+^ PBMC after coculture with neutrophils in HAE patients (60.85 %, *p* = 0.0018) and not in HD (Figure 6(e)). In CD8^+^ T-cells, surface CD25 expression was considerably reduced after coculture with neutrophils in HAE patients (52.18 %, *p* = 0.0049) and less in HD (20.11 %, *p* = 0.0297) (Figure 6(f)).

Neutrophils reduced the ratio of IFN-*γ* producing CD4^+^ PBMC both in HAE patients (71.11 %, *p* = 0.0046) and in HD (38.23 %, *p* < 0.0001) (Figure 6(g)). The ratio of IFN-*γ* producing CD8^+^ PBMC was reduced after coculture with neutrophils to a similar extent in HAE patients (42.64 %, *p* = 0.0007) and HD (35.97 %, *p* = 0.0210) (Figure 6(h)). The decreases in IFN-*γ* did not significantly differ between the patient and donor group, neither in CD4^+^ PBMC nor in CD8^+^ PBMC.

**Figure 7:**
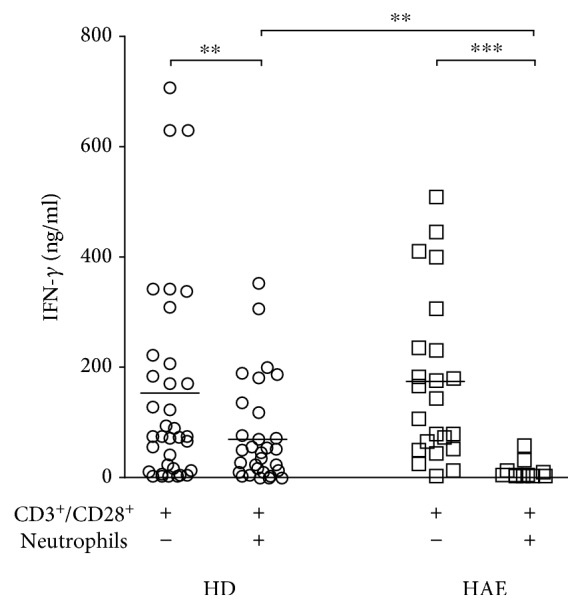
Co-incubation of T-cells with autologous neutrophils in response to stimulation with CD3/CD28 resulted in reduced IFN-*γ* accumulation in culture supernatants in response to stimulation with CD3/CD28. Horizontal bars represent medians, analyzed using Wilcoxon or Mann-Whitney *U* test as appropriate; horizontal bars represent medians: ^∗^ *p* ≤ 0.05, ^∗∗^ *p* ≤ 0.01, and ^∗∗∗^ *p* ≤ 0.001.

In addition, IFN-*γ* protein accumulation in the medium upon CD3/CD28 stimulation was lower after T-cell coculture with neutrophils from both HAE patients (*p* < 0.0001) and healthy controls (*p* < 0.006; Wilcoxon test); however, the decrease in IFN-*γ* concentration was significantly more pronounced in HAE patients (*p* < 0.002; Mann-Whitney test) (Figure 7).

## 4. Discussion

Neutrophils are known to have the potential to influence several processes preceding the formation of bradykinin, a major mediator of swelling in HAE. To date, however, only a limited number of studies have pointed to the role of neutrophil activation in HAE, all of them addressing markers in serum or plasma [15, 22–24]. Changes in neutrophil activation markers were detected mostly during HAE attacks in previous studies [17, 24, 25]. Regarding symptom-free periods, only single cytokines related to neutrophil activation (IL-8, IL-17) have been described as upregulated in individual patients so far [24, 26].

In our study, relative mRNA expression analyses showed increased *CD274*, *IL1B*, *IL1RN*, *IL8*, *MMP9*, and *TLR4* levels in patients' neutrophils compared to the neutrophils obtained from HD. Proteins coded by genes we investigated are functionally related, and their mutual interactions have been described.

IL-8 is considered the most potent neutrophil-activating chemokine. It is cleaved by MMP9 (gelatinase B), and the truncated form displays enhanced biological activities. Conversely, IL-8 triggers neutrophils to release MMP9 from their granules. MMP9 promotes neutrophil migration by degrading extracellular matrix components [27]. MMP9 is required to process another proinflammatory cytokine, IL-1*β*, to its biologically active form, and conversely, IL-1*β* induces *MMP9* expression. MMP9 and IL-1*β* often express simultaneously at sites of inflammation, where MMP9 can regulate IL-1*β* action by degrading the mature cytokine [28, 29]. Regulating IL-1*β* activity can be mediated by IL-1RN (IL-1R antagonist), which competes with IL-1 for the binding to the functional receptor [30].

IL-8 is produced by epithelial, endothelial cells and monocytes in response to bacterial infections or antigen (such as LPS) stimulation [31–33]. Although the IL-8 plasma level in HD is normally very low, the total level can be increased after stimulation [34]. However, neutrophils are not only targets for IL-8 but also its producers [35], which should enable to reach the effective number of neutrophils in inflammatory tissue by a positive feedback mechanism to be reached [36]. We detected decreased IL-8 plasma protein levels in patients compared to the healthy donors which partly corresponds to another study where IL-8 protein levels were shown to increase in HAE patients during attacks, but not in a symptom-free period compared to controls [17]. It has previously been shown that bacterial (LPS) stimulation led to increased levels of both released and neutrophil-associated IL-8, while most of the IL-8 remained cell-associated in unstimulated neutrophils [34]. As we only measured plasma IL-8 levels, it is possible that neutrophil-associated IL-8 levels were normal or increased, while plasma levels were decreased. It further supports our hypothesis that HAE patients' neutrophils are not fully activated in a symptom-free period, but they rather stay in a preactivated state, expressed at the transcriptional level, until they get some stimulus resulting in edema attack development. Additionally, we detected higher Treg levels in HAE patients than controls. Tregs are known to produce IL-10 and induce other cell types to produce IL-10 [37], and IL-10 was demonstrated to reduce IL-8 production by neutrophils stimulated with LPS, while having no significant effect on unstimulated cells [38]. We can speculate that higher IL-10 levels could aid in preventing neutrophils to become fully activated and keeping them in a preactivated state between attacks in HAE patients.

The above-mentioned examples of relationships between proteins connected to neutrophil activation could explain some of the correlations we observed at the transcript level in the HD group. The correlation analysis results suggest a balance between mRNA expression levels of the genes in the HD group. Interestingly, in the group of HAE patients, this balance seems to be disrupted. We have no explanation for the lower correlation rate observed in HAE patients, but looking at the high interindividual variability in the distribution of different genes' mRNA levels, we suppose the different mechanisms exerted in each HAE patient might contribute to this imbalance to a different extent.

In our study, we did not observe any influence on gene expression from the prophylactic drugs used. To the best of our knowledge, there are only rare reports addressing a potential effect of HAE prophylactic treatment on neutrophil activation or function in the literature. Tranexamic acid (TXA) was shown to influence neither LPS-induced neutrophil activation (CD11b, CD66b, and L-selectin expression) or degranulation [39] nor neutrophils' ability to migrate through fibrin clots [40]. On the other hand, TXA was demonstrated to repress upregulated neutrophil-recruiting chemokines CXCL1 and CXCL5 in a mouse model [41]. In our cohort, there were 8 HAE patients receiving prophylactic medication, 4 of them treated with danazol and the remaining 4 with TXA (see Supplementary table 1). Separate subanalyses showed no difference in of any of the analyzed genes' mRNA level between patients treated with either danazol or TXA and the nontreated group (data not shown). However, the number of cases was small.

The preactivated neutrophil state in HAE patients was further supported at the surface marker level using flow cytometry, where decreased CD16 and increased CD11b MFI were observed in HAE patients compared to HD. Regarding activation-specific markers, increased CD87^+^ and CD274^+^ neutrophil ratios were detected in HAE patients. On the other hand, plasma NE and MPO levels did not differ in the patients when compared to the controls. Both enzymes are stored in the neutrophils' azurophilic granules and are released following neutrophil activation. NE plasma levels were shown to be increased, e.g., in patients with pneumonia [42, 43] or inflammatory bowel disease [44, 45]. Systemic levels of MPO are elevated, e.g., in patients with pelvic inflammatory disease [46] and rheumatic arthritis [47]. All these findings indicate that HAE patients' neutrophils did not reach full activation as observed in fully developed inflammatory disorders or some pathways exerting inhibitory activity on neutrophils might be involved.

C11b (integrin alpha-M (ITGAM), complement receptor 3 (CR3)) is an integrin that functions both as an adhesive molecule enhancing diapedesis and as a C3R mediating phagocytosis or degranulation in response to iC3b-opsonized microorganisms [48]. It has been shown that CD11b is required for netosis [49]. When neutrophils are stimulated by immobilized immune complexes, CD11b and CD16 are activated and their interaction leads to the induction of NETs' formation which promotes FXII autoactivation [50]. Activating either TLR4 (corresponding transcript was upregulated in our patient group) or TLR2 is associated with CD11b upregulation on neutrophils [51, 52].

CD87 (urokinase plasminogen activator receptor (uPAR)) was originally described as a receptor for a urokinase-type plasminogen activator that facilitates converting plasminogen to plasmin [53]. Plasmin is capable of activating factor XII [54]. Activated factor XII catalyses the conversion of prekallikrein to kallikrein, which enables the cleavage of single-chain high molecular weight kininogen (HMWK) and thus generates bradykinin and two-chain HMWK [8, 55, 56].

As the C1 inhibitor is a known inhibitor of plasmin [57], decreased level and/or impaired C1 inhibitor function, together with increased neutrophil surface expression of CD87, suggests an enhanced plasmin formation contributes to HAE types I and II. The role of the fibrinolytic system has already been reported in some HAE III cases. Four mutations in the *FXII* were identified, leading to increased mutant FXII sensitivity to plasmin cleavage [58–60]. Plasmin's importance in HAE pathogenesis was further emphasized by a recently described new form of HAE with a normal C1 inhibitor level and function caused by a mutation in the *PLG* gene [6].

Taken together, neutrophils' alertness demonstrated at the transcript level was supported by increased activation markers right on their surface or by increased CD274^+^ and CD87^+^ cell ratios, but not by elevated NE, MPO, or IL-8 plasma levels in HAE patients compared to controls. Neutrophils' activation during edema attacks has been documented earlier [17, 24, 25]. Based on our results, we suggest that neutrophils are in an alerted, preactivated state in HAE patients, even in a symptom-free period. The C1 inhibitor is well known for regulating complement, contact (kallikrein-kinin), coagulation, and fibrinolytic systems by interacting with C1s, C1r, MASP-1, MASP-2, factor XIIa, factor XIa, kallikrein, and plasmin. However, The C1 inhibitor serves as a multipotent anti-inflammatory agent, independent of its protease inhibitory function. It inhibits LPS binding to the LPS receptor complex on the macrophages' surface, thereby suppressing TNF-alpha and other inflammatory mediators' production [61, 62]. Indeed, administering the C1 inhibitor in patients with endotoxemia and in mice with experimentally induced sepsis resulted in inflammatory response attenuation and improved survival, respectively [63, 64]. Thus, a lack of functional C1 inhibitor in HAE patients may lead to transcriptional dysregulation of genes coding for molecules playing an important role in inflammation, which may become expressed at protein level during an edema attack after getting some stimulus.

To assess whether an altered state of patients' neutrophils is reflected also at the functional level, we analyzed their potential suppressive effect on T-cells/PBMC. Accumulating evidence supports the role of specific neutrophil subsets in the suppression of T-cell functions. Such negative regulation is important for preventing excessive tissue damage during inflammation, but it can be detrimental in various pathological conditions [65, 66]. One of the neutrophil-mediated T-cell suppression mechanisms consists of a local hydrogen peroxide release into the immunological synapse between neutrophil and T-cell, requiring CD11b upregulation [23]. CD274 (PD-L1) upregulation on neutrophils with subsequent interaction with PD-1 on T-cells was reported to mediate T-cell suppression too [18]. Thus, CD11b and CD274 upregulation on neutrophils suggests a presence of neutrophils potentially suppressing T-cells in the HAE patients involved in our study. This was further supported by the functional assay results evaluating neutrophils' effect on T-cell activation. Neutrophils' suppressive function added to the T-cells was manifested as a decrease in the ratio of CD25^+^ and INF-*γ* ^+^ T-cells and a more pronounced decrease in IFN-*γ* production in HAE patients. The fact that the decrease was observed in PBMC too suggests that the neutrophils' suppressive activity was not negated by monocytes' stimulative effect.

HAE patients often have reduced C2 and C4 complement components, leading to decreased immunocomplex solubilisation [67] but also polyclonal B-cell activation [68], which may predispose them to autoimmune diseases. Although several studies pointed to a possible increase in various autoantibodies in HAE patients [68, 69], the prevalence of autoimmune diseases in HAE patients does not seem to be elevated [70, 71]. This was well documented in female HAE patients with a higher prevalence of antithyroid autoantibodies but no increase in spontaneous overt hypothyroidism [72]. It could be hypothesized that the neutrophil-mediated T-cell suppression indicated in our study may prevent HAE patients from autoimmune disease development, even in the presence of autoantibodies. This hypothesis is further supported by detecting an increased Treg subpopulation in HAE patients compared to controls. Nevertheless, it has previously been shown that Treg cells may suppress not only autoimmune but also anti-infection response, mainly in chronic infections [73]. Thus, our results of the neutrophils' increased regulatory effect on T-lymphocyte function and increased Treg cell population in HAE patients raise the question whether these mechanisms cannot induce some degree of immunodeficiency. There is no data supporting the increase in frequency of acute or chronic infections in HAE patients. Although chronic infections like *Helicobacter pylori* [74] or bacteriuria [75] were shown to increase HAE attack frequency, a direct comparison of their occurrence between HAE patients and the healthy population was missing from these studies. Only one study from Poland did not demonstrate an increased antibody frequency against *H. pylori* compared to the healthy population both in children and in adult HAE patients [76]. It is obvious that more detailed immune regulatory mechanism studies in HAE patients would be required to confirm this hypothesis.

In summary, the results of the presented study suggest a preactivated state and dysregulation of neutrophils at the transcript level in HAE patients even in a symptom-free period, which might make them more susceptible to edema formation. Furthermore, neutrophils' T-cell suppressive capacity was shown, raising the question of their possible role in preventing autoimmune diseases in HAE patients. Although the results need to be confirmed at the protein level and in a larger cohort of patients in different populations, the observations presented here provide a novel view on neutrophils' potential role in HAE pathogenesis.

## Figures and Tables

**Table 1a:** (a) HAE

	*IL8*	*IL1B*	*IL1RN*	*MMP9*	*TLR4*	
*CD274*	∗∗∗ 0.65	ns 0.40	ns 0.26	∗∗ 0.61	∗∗ 0.60	*p* *r_s_*

*IL8*		ns 0.19	∗∗ 0.63	ns 0.14	∗∗∗ 0.65	*p* *r_s_*

*IL1B*			ns 0.19	∗ 0.50	ns 0.04	*p* *r_s_*

*IL1RN*				ns 0.29	ns 0.37	*p* *r_s_*

*MMP9*					ns *-0.19*	*p* *r_s_*

**Table 1b:** (b) HD

	*IL8*	*IL1B*	*IL1RN*	*MMP9*	*TLR4*	
*CD274*	∗∗∗ 0.63	∗∗∗ 0.63	ns 0.30	∗∗∗ 0.55	∗∗∗ 0.67	*p* *r*_s_

*IL8*		∗∗∗ 0.92	ns 0.04	∗∗∗ 0.59	∗∗∗ 0.73	*p* *r*_s_

*IL1B*			ns -0.10	∗∗∗ 0.56	∗∗∗ 0.70	*p* *r*_s_

*IL1RN*				ns 0.19	ns 0.08	*p* *r*_s_

*MMP9*					∗∗∗ 0.60	*p* *r*_s_

*p*: *p* value: ^∗^ *p* ≤ 0.05, ^∗∗^ *p* ≤ 0.01, and ^∗∗∗^ *p* ≤ 0.001; *r*_*s*_: Spearman's rank correlation coefficient.

## Data Availability

All data used to support the findings of this study are included within the article or within the supplementary file.

## Supplementary Materials

Supplementary 1 Table S1: complement levels and function, disease type, and treatment in HAE patients.

Supplementary 2 Fig. S1: the relative mRNA expression of genes related to neutrophil activation in patients with hereditary angioedema (HAE) compared to healthy donors (HD) (horizontal bars represent medians; ns: nonsignificant; data analyzed using Mann-Whitney *U* test). Fig. S2: (A) gating strategy for phenotypic characterization of isolated neutrophil population from isolated neutrophils. The reduced PD-L1 and CD11b expression in purity neutrophils after isolation is shown in grey. Representative data from one of 39 healthy donors. (B) Gating strategy for phenotypic characterization of neutrophil population in fresh blood. Representative data from one of 39 healthy donors. Fig. S3: neutrophils were gated as a CD45^+^CD15^+^CD16^+^ SSC^high^ cells. (A, B) Increased expression of CD11b (A) and PD-L1 (B) on neutrophils in fresh blood of HAE patients. (C) Decreased CD16 levels in fresh blood of HAE patients, representative examples, and cumulative data. Fig. S4: neutrophil surface CD80 expression in HAE patients (HAE) compared to healthy donors (HD) (ns: nonsignificant; MFI: median fluorescence intensity; horizontal bars represent medians; data analyzed using Mann-Whitney *U* test). Fig. S5: gating strategy for the analysis of CD25 expression/IFN-*γ* production on/in T-cells. Representative data from one of 20 HAE patients. Fig. S6: decreases in CD25 expression/IFN-*γ* production on/in T-cell subpopulations after coculture with neutrophils. Representative data from one of 20 HAE patients.
