# Supplementary material for: Neutrophils Are Dysregulated in Patients with Hereditary Angioedema Types I and II in a Symptom-Free Period
Source: Mediators Inflamm. 2019 May 19;2019:9515628. doi: 10.1155/2019/9515628 (PMC6545807; doi:10.1155/2019/9515628)
Supplement: Supplementary 1 — Table S1: complement levels and function, disease type, and treatment in HAE patients. [file 9515628.f1.pdf]

Supplementary table 1: Complement levels and function, disease type and treatment in HAE patients

patient ID		CH50	AH50	C1INH	C1INH function	C1q	C2	C5	C3	C4	HAE type	Treatment
	units	%	%	mg/L	%	mg/L	mg/L	mg/L	g/L	g/L		
	normal range	>70	>30	210-390	>68	100-250	10-30	80-170	0.79-1.52	0.10-0.38		
1		73	118	**169**	**26**	205	13.8	**180**	1.05	**0.07**	1	-
2		94	101	378	69	190	13.2	**225**	0.89	**0.08**	2	danazol
3		**62**	82	432	**60**	193	19.0	**202**	1.12	**0.05**	2	tranexamic acid
4		100	100	**95**	**38**	201	17.3	**211**	0.84	**0.08**	1	-
5		95	87	**95**	71	231	17.0	161	0.94	0.11	1	danazol
6		**67**	89	**48**	**36**	212	15.4	158	**0.76**	**0.04**	1	-
7		101	118	**166**	**43**	182	15.9	**203**	1.43	0.11	1	-
8		**60**	38	**75**	**52**	166	19.2	138	0.98	**0.06**	1	-
9		108	95	**99**	**54**	215	16.5	**215**	1.05	**0.09**	1	danazol
10		**25**	89	**44**	**26**	141	13.5	168	0.95	**0.02**	1	-
11		**32**	104	**62**	**52**	181	13.4	157	0.82	**0.02**	1	danazol
12		131	81	**66**	**66**	197	22.1	122	**0.68**	**0.07**	1	-
13		93	109	**207**	**18**	203	25.0	**225**	1.57	0.11	1	-
14		125	99	**190**	**40**	196	16.3	**224**	1.19	**0.08**	1	-
15		71	61	660	**29**	194	11.2	164	**0.75**	**0.07**	2	-
16		91	89	**73**	**40**	194	12.7	154	0.88	0.10	1	-
17		85	94	**57**	**41**	182	18.2	159	1.02	**0.05**	1	tranexamic acid
18		**41**	29	**33**	**32**	157	13.5	148	0.80	**0.03**	1	tranexamic acid
19		**21**	64	**55**	**50**	207	12.1	164	**0.68**	**0.04**	1	-
20		**64**	81	**54**	**26**	199	17.7	**219**	1.10	**0.05**	1	-
21		**12**	107	**36**	**30**	122	**8.8**	135	0.96	**0.03**	1	-
22		**57**	80	**48**	**51**	207	19.0	**195**	0.89	**0.06**	1	-
23		**1**	64	**34**	**18**	202	**4.1**	**238**	0.88	**0.02**	1	tranexamic acid

Legend: Values out of normal range are marked in bold.
