# Supplementary material for: Neutrophils Are Dysregulated in Patients with Hereditary Angioedema Types I and II in a Symptom-Free Period
Source: Mediators Inflamm. 2019 May 19;2019:9515628. doi: 10.1155/2019/9515628 (PMC6545807; doi:10.1155/2019/9515628)
Supplement: Supplementary 2 — Fig. S1: the relative mRNA expression of genes related to neutrophil activation in patients with hereditary angioedema (HAE) compared to healthy donors (HD) (horizontal bars represent medians; ns: nonsignificant; data analyzed using Mann-Whitney U test). Fig. S2: (A) gating strategy for phenotypic characterization of isolated neutrophil population from isolated neutrophils. The reduced PD-L1 and CD11b expression in purity neutrophils after isolation is shown in grey. Representative data from one of 39 healthy donors. (B) Gating strategy for phenotypic characterization of neutrophil population in fresh blood. Representative data from one of 39 healthy donors. Fig. S3: neutrophils were gated as a CD45+CD15+CD16+ SSChigh cells. (A, B) Increased expression of CD11b (A) and PD-L1 (B) on neutrophils in fresh blood of HAE patients. (C) Decreased CD16 levels in fresh blood of HAE patients, representative examples, and cumulative data. Fig. S4: neutrophil surface CD80 expression in HAE patients (HAE) compared to healthy donors (HD) (ns: nonsignificant; MFI: median fluorescence intensity; horizontal bars represent medians; data analyzed using Mann-Whitney U test). Fig. S5: gating strategy for the analysis of CD25 expression/IFN-γ production on/in T-cells. Representative data from one of 20 HAE patients. Fig. S6: decreases in CD25 expression/IFN-γ production on/in T-cell subpopulations after coculture with neutrophils. Representative data from one of 20 HAE patients. [file 9515628.f2.pdf]

Neutrophils are activated in patients with hereditary angioedema type I and II in a symptom-free period

Supplementary material

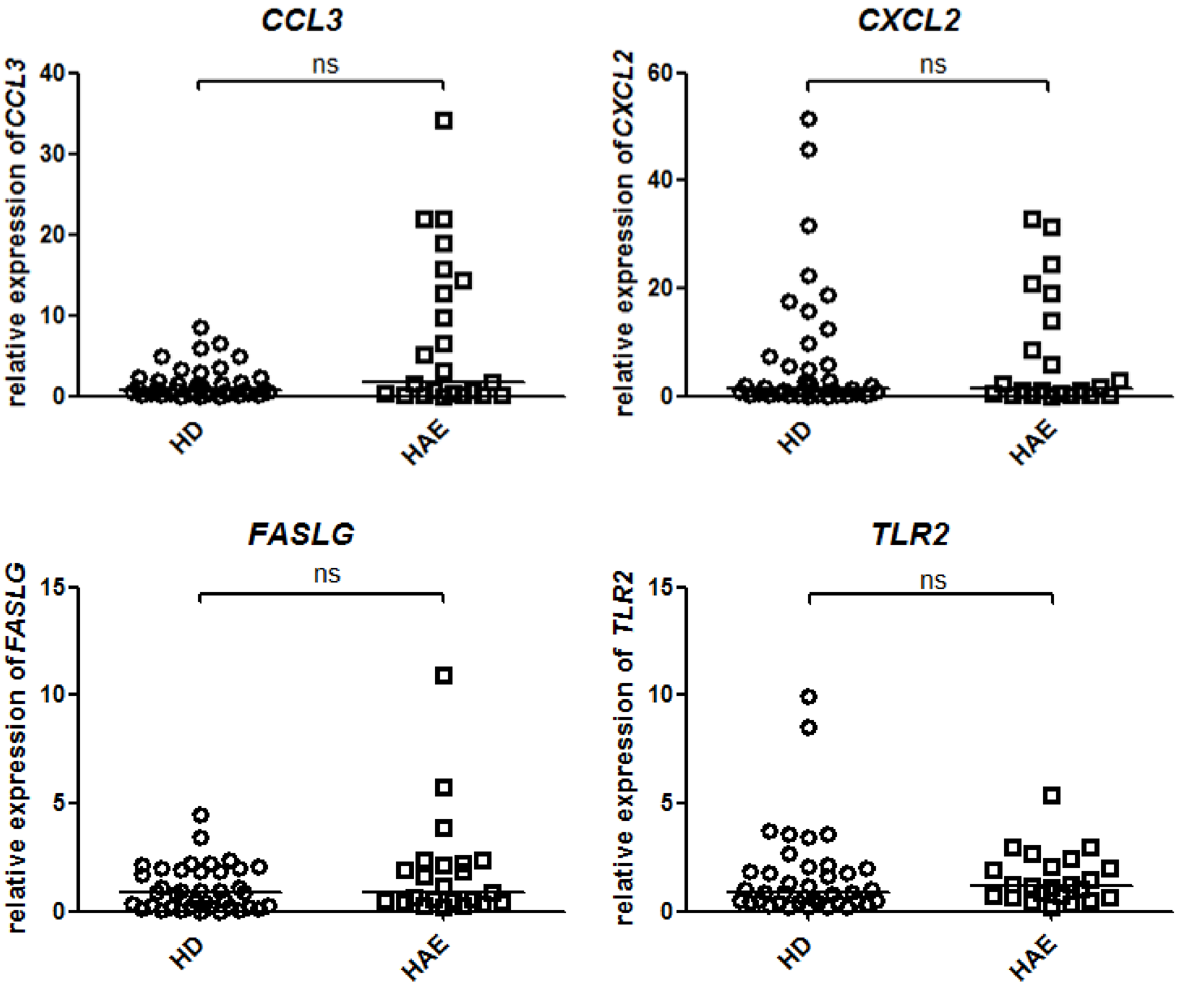

Fig. S1. The relative mRNA expression of genes related to neutrophil activation in patients with hereditary angioedema (HAE) compared to healthy donors (HD). (horizontal bars represent medians; ns - non-significant; data analyzed using Mann-Whitney U test)

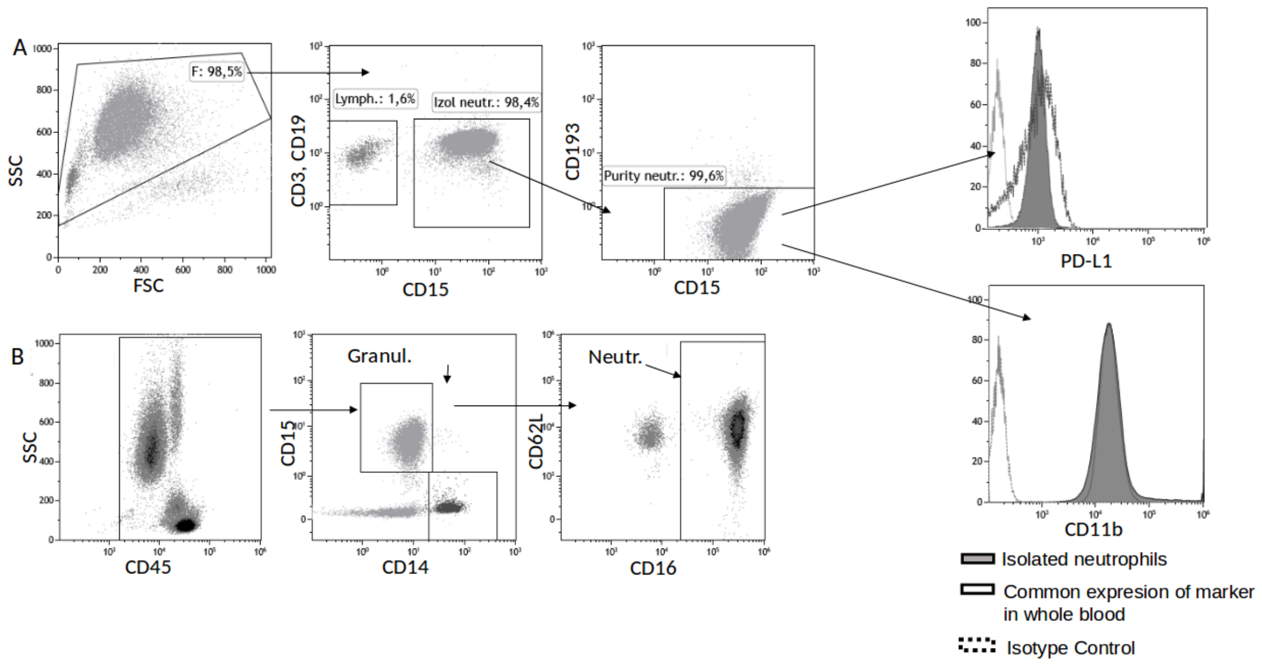

Fig. S2. (A) Gating strategy for phenotypic characterization of isolated neutrophil population from isolated neutrophils. The reduced PD-L1 and CD11b expression in purity neutrophils after isolation is shown in grey. Representative data from one of 39 healthy donors. (B) Gating strategy for phenotypic characterization of neutrophil population in fresh blood. Representative data from one of 39 healthy donors.

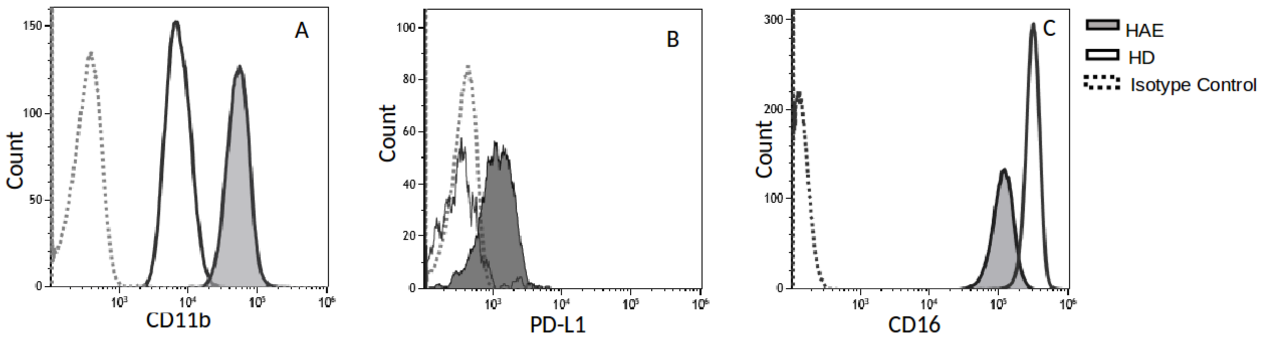

Fig. S3. Neutrophils were gated as a $CD45^{+}CD15^{+}CD16^{+}$ SSC^{high} cells. A, B) Increased expression of CD11b (A) and PD-L1 (B) on neutrophils in fresh blood of HAE patients. C) Decreased CD16 levels in fresh blood of HAE patients; representative examples, cumulative data.

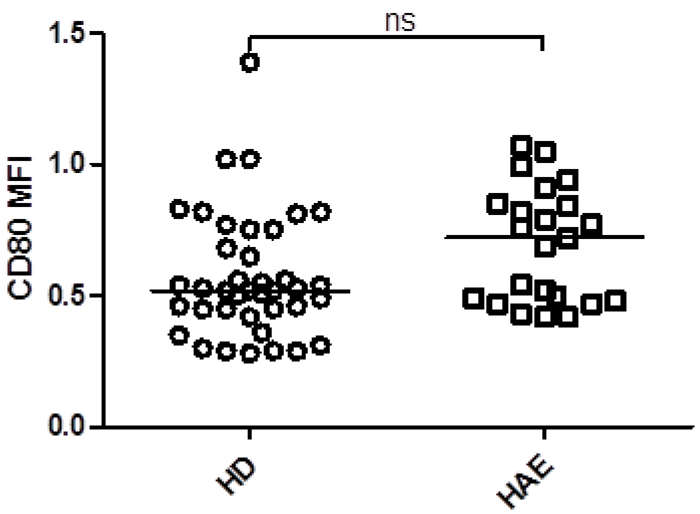

Fig. S4. Neutrophil surface CD80 expression in HAE patients (HAE) compared to healthy donors (HD). (ns - non-significant; MFI - median fluorescence intensity; horizontal bars represent medians; data analyzed using Mann-Whitney U test)

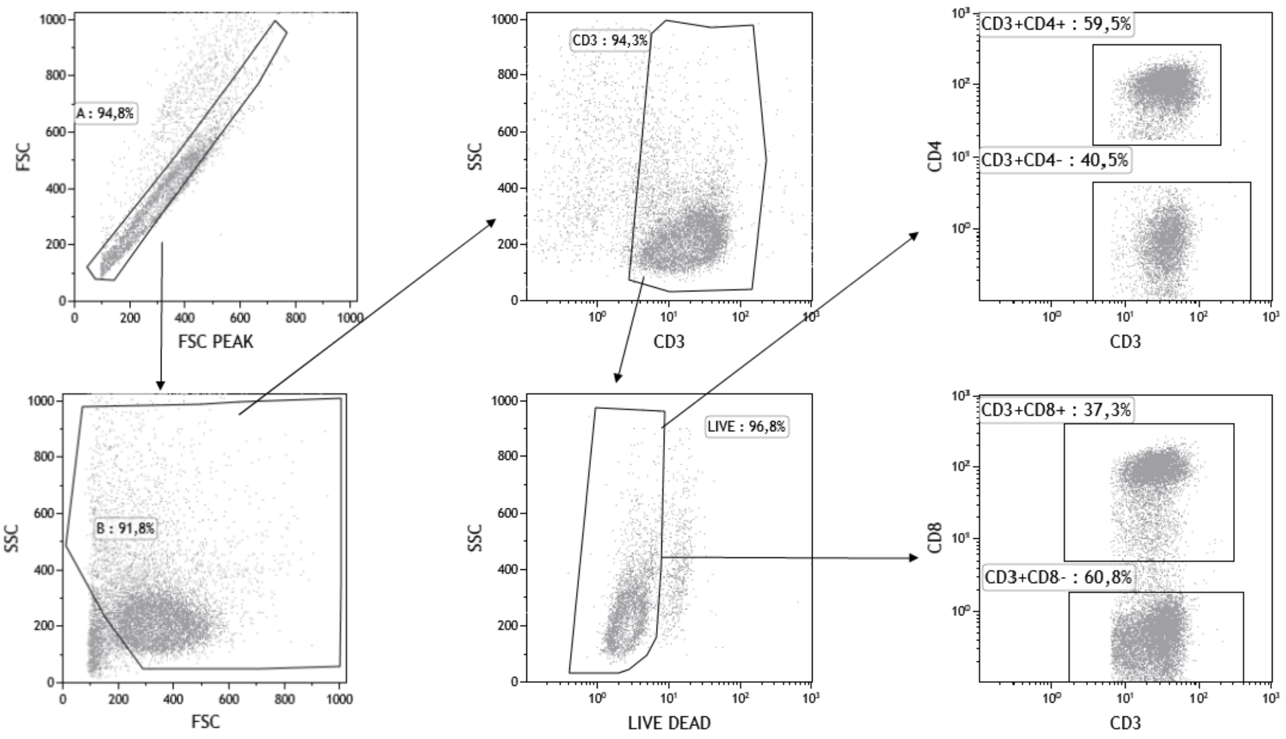

Fig. S5. Gating strategy for the analysis of CD25 expression/IFN-γ production on/in T-cells. Representative data from one of 20 HAE patients.

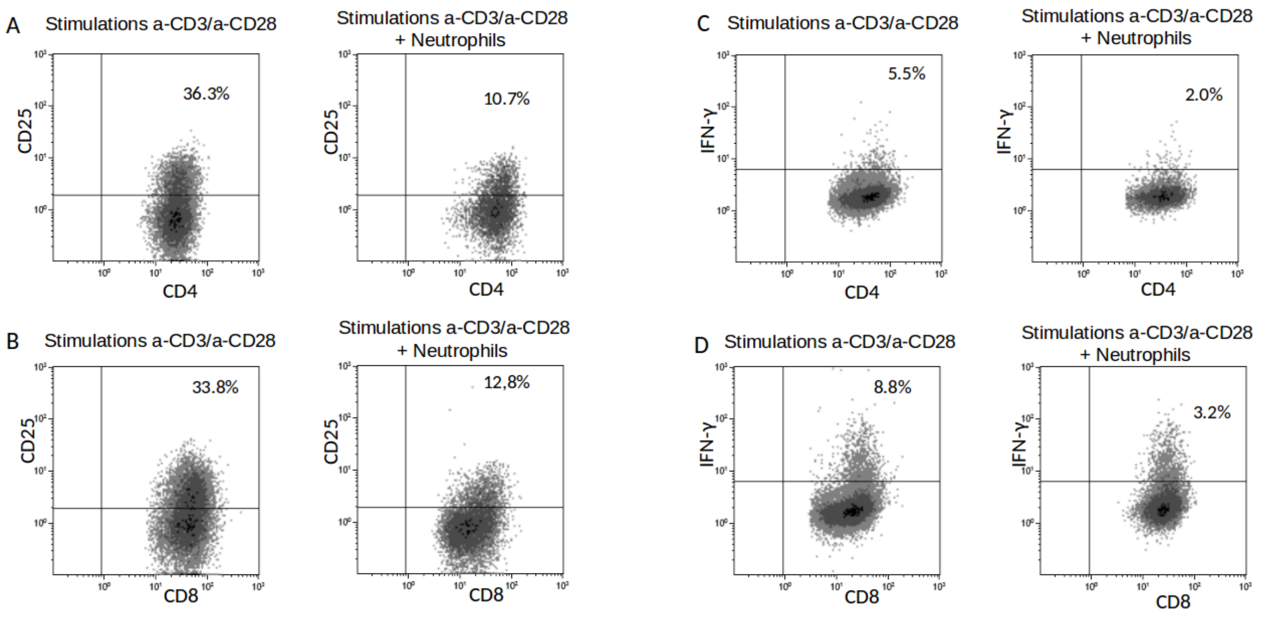

Fig. S6. Decreases in CD25 expression/IFN-γ production on/in T-cell subpopulations after co-culture with neutrophils. Representative data from one of 20 HAE patients.
